# Lack of serologic evidence for an association between Cache Valley Virus infection and anencephaly and other neural tube defects in Texas.

**DOI:** 10.3201/eid0302.970215

**Published:** 1997

**Authors:** J F Edwards, K Hendricks

**Affiliations:** Texas A&M University, College Station, USA.

## Abstract

We tested the hypothesis that Cache Valley Virus (CVV), an endemic North American bunyavirus, may be involved in the pathogenesis of human neural tube defects. This investigation followed a 1990 and 1991 south Texas outbreak of neural tube defects with a high prevalence of anencephaly and the demonstration in 1987 that in utero infection by CVV was the cause of outbreaks of central nervous system and musculoskeletal defects in North American ruminants. Sera from 74 women who gave birth to infants with neural tube defects in south Texas from 1993 through early 1995 were tested for CVV neutralizing antibody. All tested sera did not neutralize CVV. These data suggest that CVV is not involved in the induction of human neural tube defects during nonepidemic periods but do not preclude CVV involvement during epidemics. Other endemic bunyaviruses may still be involved in the pathogenesis of neural tube defects or other congenital central nervous system or musculoskeletal malformations.


### Lack of Serologic Evidence for an Association between Cache Valley Virus Infection and Anencephaly and other Neural Tube Defects in Texas

We tested the hypothesis that Cache Valley Virus (CVV), an endemic North American bunyavirus, may be involved in the pathogenesis of human neural tube defects. This investigation followed a 1990 and 1991 south Texas outbreak of neural tube defects with a high prevalence of anencephaly and the demonstration in 1987 that in utero infection by CVV was the cause of outbreaks of central nervous system and musculoskeletal defects in North American ruminants. Sera from 74 women who gave birth to infants with neural tube defects in south Texas from 1993 through early 1995 were tested for CVV neutralizing antibody. All tested sera did not neutralize CVV. These data suggest that CVV is not involved in the induction of human neural tube defects during nonepidemic periods but do not preclude CVV involvement during epidemics. Other endemic bunyaviruses may still be involved in the pathogenesis of neural tube defects or other congenital central nervous system or musculoskeletal malformations.

Anencephaly, spina bifida, and encephalocele (the major types of neural tube defects) are generally due to the failure of the neural tube to close during early embryonic development (1). Neural tube defects are among the most common and most severe major birth defects. Anencephaly is caused by failure of the anterior neuropore to close during embryonic development and results in total or partial absence of the cranial vault, the covering skin, and the brain. Infants with anencephaly are stillborn or die shortly after birth. Spina bifida is caused by a disturbance in the normal closure of neural walls and results in spinal cord defects. Most infants with spina bifida survive surgical repair of the defect with residual neurologic handicaps of varying severity. Encephaloceles are skull defects through which skin-covered meninges, with or without brain tissue, herniate. Small to moderately sized encephaloceles are surgically correctable (1-4).

The prevalence of neural tube defects in the United States has been steadily declining (5) and is currently estimated to be six per 10,000 births. The prevalence of anencephaly in the United States has likewise declined and is now approximately three per 10,000 live births (4). A vital record study of anencephaly in Texas showed that from 1981 to 1986 the prevalence rate was 3.8 to 4.3 cases per 10,000 births (6). This study also showed that in south Texas during this period the average annual prevalence of anencephaly was approximately 4.9 per 10,000 births. Women with Hispanic surnames, three or more previous live births, history of stillbirth, or residence in east or south Texas were at increased risk for neural tube defect–affected pregnancies. On the basis of vital record data, the annual prevalence of anencephaly in south Texas from 1981 through 1986 was approximately 4.9 per 10,000 live births.

In 1991 three babies with anencephaly were born over a 36-hour period at a single hospital in Cameron County, the southernmost county in Texas (Brownsville is the county's largest city). The ensuing study, which used active multisource case finding rather than vital records, showed that the neural tube defect prevalence rate increased from 14.7 per 10,000 births in 1986 to 1989 to 27.1 per 10,000 births in 1990 to 1991. The higher rate was due largely to an increase in anencephaly cases. From 1986-89 to 1990-91, the average annual anencephaly prevalence rate rose from 9.6 to 19.7 per 10,000 births (7,8).

Despite the high prevalence of neural tube defects and the significant rate of illness associated with them, much remains to be learned about their complex multifactorial etiology. Evidence suggests that these defects are etiologically heterogeneous and may follow fetal insults such as maternal diabetes, hyperthermia, folic acid deficiency, and anticonvulsant (valproate) therapy (9-13).

In 1987, before the increase in prevalence of human anencephaly in south Texas, an outbreak of severe congenital malformations of the central nervous system and musculoskeletal system of lambs occurred in San Angelo, Texas (14). At the time of the outbreak, there was no active surveillance of human birth defects in San Angelo, and no reports were received of human birth defects in the area. The ovine problem was later found to be caused by in utero infection by Cache Valley Virus (CVV). Although this insect-borne bunyavirus had been known to commonly infect North American ruminants (15), it was not thought to be of clinical significance.

Experimentally, it was determined that CVV infection of the dam in early gestation and transplacental infection of the ovine fetus could produce severe brain malformations and arthrogryposis multiplex congenita, an anomaly characterized by limbs fixed in contracture (14). Central nervous system malformations associated with experimental and spontaneous CVV infection include hydrocephalus, hydranencephaly, porencephaly, micrencephaly, and micromelia. After the syndrome was characterized, outbreaks of CVV-induced malformations in ruminants were diagnosed throughout North America, and work by Calisher and Sever (16) also linked CVV to congenital cases of human macrocephaly in the United States.

Other bunyaviruses can cause identical congenital malformations of the central nervous system in experimentally infected livestock (14). Human congenital morbidity has also been correlated with maternal antibody to bunyaviruses (16). A recent study correlated both human microcephaly and macrocephaly with antibody to Tenshaw virus in mothers of infants with these illnesses.

We decided to test the hypothesis that CVV infection was related to human neural tube defects. Public concern regarding the 1990 to 1991 cluster in Brownsville, Texas, (7) had resulted in an ongoing project in the 14 Texas counties that border Mexico. A neural tube defect surveillance and folic acid intervention project were implemented in 1993, and a case-control study was begun in mid-1995. Sera from case patients had been banked before the case-control study began.

Sera from 74 women who lived in the Texas border counties and had neural tube defect–affected pregnancies (36 with spina bifida, 34 with anencephaly, and 4 with encephalocele) from 1993 through early 1995 were examined for a possible link between CVV and neural tube defects. With a standard microtiter serum dilution neutralization test (17), the sera were screened at final dilutions of 1:2, 1:4, 1:8, and 1:16. The virus used in all tests was the prototype CVV (strain 6V-633, provided by the Centers for Disease Control and Prevention [CDC], Ft. Collins, Colorado), which had been passaged one time in Vero cells after receipt from CDC. Controls included sera from women of undetermined CVV status who gave birth to healthy infants in south Texas [8]; sera collected from sheep before CVV infection [3]; and normal macaque [4], horse [1], and bovine [1] sera. Positive controls included CVV–convalescent-phase ovine sera [3] and CVV antibody–positive sera from a horse and a cow. No serum neutralization activity for CVV was detected in sera from women who gave birth to healthy infants or infants with neural tube defects. Had CVV infection been present in these women during gestation, CVV antibody would have been detectable postpartum.

Before this study, the relationship between CVV and human neural tube defects was unknown. Testing an adequate number of controls is critical when seroepidemiology is used to establish a causal relationship between an agent and a low frequency event or malformation, particularly when case patients have evidence of antibodies against the agent of interest. In this study, there was no evidence that CVV was related to the neural tube defect cases observed in Texas from 1993 through early 1995. Had CVV antibodies been detected in serum from women with neural tube defect–affected pregnancies, it would have been necessary to test control sera from age- and location-matched women with normal births.

The average annual neural tube defect prevalence rate in Cameron County, Texas, for 1992 to 1995 has returned to the 1986 to 1989 rate of approximately 14-15 cases per 10,000 births. These data suggest that CVV is not involved in the induction of human neural tube defects during nonepidemic periods but do not preclude CVV involvement during epidemics. CVV may still be involved in induction of other human malformations. Other endemic bunyaviruses may be involved in the pathogenesis of neural tube defects and of other congenital nervous system or musculoskeletal malformations (18,19). It would seem valid to continue to investigate the relationship of CVV and other arboviruses to human developmental illness and death rate. Because of the wide variety of defects caused by these viruses, laboratory models of fetal infection by the Bunyaviridae would facilitate the understanding of viral teratogenesis mechanisms in humans.

**J. F. Edwards* and K. Hendricks†**
*Texas A&M University, College Station, Texas, USA; and †Texas Department of Health, Austin, Texas, USA

### References

1. Copp AJ, Brooks SA, Estibeiro JP, Shum AS, Cockcroft DL. The embryonic development of mammalian neural tube defects. Prog Neurobiol 1990;35:363-403.
2. World Health Organization. Congenital malformations worldwide: a report from the international clearinghouse for birth defects monitoring systems 1991. New York: Elseviers Science Publishers, 1991:41-79.
3. Campbell LR, Dayton DH, Sohal GS. Neural tube defects: a review of human and animal studies on the etiology of neural tube defects. Teratology 1986;34:171-87.
4. Thomas JA, Markovac J, Ganong WF. Anencephaly and other neural tube defects. Front Neuroendocrinol 1994;15:197-201.
5. Yen IH, Khoury MJ, Erickson JD, James LM, Waters GD, Berry RJ. The changing epidemiology of neural tube defects: United States. Am J Dis Child 1992;146:857-61.
6. Brender JD, Carmichael L, Preece MJ, Larimer GC, Suarez L. Epidemiology of anencephaly in Texas, 1981-1986. Tex Med 1989;85:33-5.
7. Albrecht LJ. Mystery in Cameron County. Physicians and health officials search for cause of high rate of anencephaly in the Rio Grande Valley. Tex Med 1994;90:16-18.
8. Texas Department of Health/Centers for Disease Control. An investigation of a cluster of neural tube defects in Cameron County, Texas. July 1, 1992.
9. Ardinger HH, Atkins JF, Blackston RD, Elsas LJ, Clarren SK, Livingstone S, et al. Verification of the fetal valproate phenotype. Am J Med Genet 1988;29:171-85.
10. Finnell RH, Taylor LW, Bennett GD. The impact of maternal hyperthermia on morphogenesis: clinical and experimental evidence for a fetal hyperthermia phenotype. Developmental Brain Dysfunction 1993;6:199-209.
11. Mills JL, Baker L, Goldman S. Malformations in infants of diabetic mothers occurs before the seventh gestational week. Diabetes 1979;28:292-3.
12. Sandford MK, Kissling GE, Joubert PE. Neural tube defect etiology: new evidence concerning maternal hyperthermia, health and diet. Dev Med Child Neurol 1992;34:661-75.
13. Willett WC. Folic acid and neural tube defects: can't we come to closure. Am J Pub Health 1992;82:666-8.
14. Edwards JF. Cache Valley virus. Vet Clin North Am: Food Anim Pract 1994;10:515-24.
15. Calisher CH, Francy DB, Smith GC, Muth DJ, Lazuick JS, Karabatsos N, et al. Distribution of Bunyamwera serogroup viruses in North America, 1956-1984. Am J Trop Med Hyg 1986;35:429-43.
16. Calisher CH, Sever JL. Are North American Bunyamwera serogroup viruses etiologic agents of human congenital defects of the central nervous system? Emerg Inf Dis 1995;1:147-51.
17. Pantuwatana S, Thompson WH, Watts DM, Hanson RP. Experimental infection of chipmunks and squirrels with LaCrosse and trivittatus viruses and biological transmission of LaCrosse virus by *Aedes triseriatus*. Am J Trop Med Hyg 1972;21:476-81.
18. Hall JG. Genetic aspects of arthrogryposis. Clin Orthop 1985;184:44-53.
19. Davies-Wynne R, Lloyd-Roberts GC. Arthrogryposis multiplex congenita; search for prenatal factors in 66 sporadic cases. Arch Dis Child 1976;51:618-23.
